# The effects of topical erythropoietin on non-surgical treatment of periodontitis: a preliminary study

**DOI:** 10.1186/s12903-021-01607-y

**Published:** 2021-05-06

**Authors:** Hoori Aslroosta, Siamak Yaghobee, Solmaz Akbari, Negar Kanounisabet

**Affiliations:** Department of Periodontics, School of Dentistry, Faculty of Dentistry, Tehran University of Medical Sciences, North Kargar Street, Tehran, Iran

**Keywords:** Non-surgical periodontal treatment, Erythropoietin (EPO), Periodontitis

## Abstract

**Background:**

The purpose of periodontal treatments is to reduce inflammation, restore gingival health and clinical attachment level gain by controlling microbial plaque formation and other etiological factors. One of the drugs that has been tested in many areas and shown good anti-inflammatory properties is erythropoietin (EPO). We evaluated the effect of this drug on the improvement of periodontitis after the phase I treatment.

**Methods:**

This study was conducted on 30 patients with stage III periodontitis who had at least two bilateral teeth with CAL of ≥ 5 mm and PPD ≥ 6 mm at ≥ 2 non‐adjacent teeth and bleeding on probing. After oral hygiene instruction and scaling and root planning (SRP), EPO gel containing a solution of 4000 units was applied deeply in the test group and placebo gel was deeply administered in the control pockets (5 times, every other day). The clinical parameters of the plaque index (PI), gingival index (GI), clinical attachment level (CAL), probing depth (PD) and bleeding index (BI) were measured at baseline and after three months of follow up. The *P*-value was set at 0.05.

**Results:**

All clinical variables improved after treatment in both groups. The BI and GI scores (which reflects the degree of gingival inflammation) showed statistically more reduction in test group. The CAL decreased from 5.1 ± 4.1 to 3.40 ± 2.71 mm; and 5.67 ± 4.32 to 4.33 ± 3.19 mm in test and control group, respectively (*P* < 0.00). After the treatment, there was a significant greater reduction in CAL and also PD values in test group (*P* < 0.01).

**Conclusion:**

Local application of EPO gel in adjunct to SRP can improve clinical inflammation and CAL gain in periodontitis.

*Trial registration*: This study was registered at 2017-11-06 in IRCT. All procedures performed in this study were approved with ID number of IR.TUMS.DENTISTRY.REC.1396.3139 in Tehran University of medical science.

**Supplementary Information:**

The online version contains supplementary material available at 10.1186/s12903-021-01607-y.

## Background

Periodontal disease is one of the most common infectious diseases associated with dental plaque accumulation. Periodontitis may lead to tooth loss and disability and it negatively affects chewing function and aesthetics [1]. Therefore accurate diagnosis and treatment of this disease is essential and based on reduction or elimination of local factors, risk management and elimination of the harmful effects of disease [2]. Periodontal deterioration occurs as a result of both direct (due to microbial invasion) and indirect tissue destruction (due to host defense response). The diffusion of bacterial products (antigens, lipopolysaccharide) across the junctional epithelium stimulate the host immune-inflammatory response in the tissues that is the major factor in the periodontal tissue degradation [3–5].

Some studies introduced effectiveness of adjunctive pharmacotherapies in the management of periodontitis which particularly targets the host immune response and a wide variety of drugs are under research for evaluating their ability to modulate destructive components of the host response [6–8].

Local drug delivery system, is one of such commonly used practices [9]. Local administration of anti-infective agents (e.g. Doxycycline), directly into the periodontal pocket, has the potential to provide greater concentrations of drug into the infected area and to reduce possible systemic side effects [9, 10].

Erythropoietin (EPO) is a hormone produced by kidneys with modifying effects on the host immune system. Contrary to the past speculation that EPO was only effective in the development of erythropoiesis, today, EPO is recognized for its regenerative properties in acute and chronic tissue damage [11, 12]. EPO attenuates both primary and secondary injury, as well as facilitates healing and restoration of function by local down-regulation of inflammatory processes triggered by injury [11]. In a study by Strunk et al. [13], investigators observed that IL-2, -6, -8, c-interferon (IFN-c) and TNF-α stimulated by lipopolysaccharide (LPS) could be significantly inhibited by rh-EPO. So, a beneficial role for administering exogenous EPO could be to rescue cells at risk of apoptosis within the penumbra by signaling through its already expressed receptor [14, 15]. In addition, EPO plays a role in invoking stem cells [16] and in improving blood supply to surrounding tissues as a protective molecule for vascular endothelial cells, which undergo apoptosis in hypoxic conditions [17]. By increasing the proliferation of stem cells, including dermal stem cells, EPO is effective in skin restorations [18–20]. In a study on mice with heat damage, treatment with EPO was associated with improved and accelerated wound healing by increasing vascular proliferation, extracellular matrix maturation, angiogenesis and vascular density [20]. Recent studies have shown the effects of EPO on the recovery of damaged nerves by increasing vascular proliferation [21]. Also, topical application of EPO improves palatal wound healing during the third and fourth weeks after free gingival graft procedures [22]. Although systemic administration of EPO have been reported to result in improved recovery of neuronal structures following ischemic neuronal damage [23, 24], this administration route was mainly studied in animal models. Human clinical studies are ongoing to confirm the safety of systemic application of EPO.

Despite the recognized properties of anti-inflammatory, anti-apoptotic, and angiogenic properties of EPO, and the role of the host response in pathogenesis of periodontal diseases, there are no studies on the use of this hormone in periodontal treatments. So, we decided to evaluate the effects of local application of EPO on the clinical outcomes of non-surgical treatment of periodontitis.

## Methods

### Study group and research design

This triple blinded randomized clinical trial conducted at the Periodontology Department of School of Dentistry at Tehran University of medical sciences. Thirty patients with stage III periodontitis were selected from the patients referred to the Periodontics department. All procedures performed in this study were approved on 06/11/2017 with ID number of IRCT2017091636203N1 in Tehran University of medical sciences. Procedures were in accordance with the ethical standards from the last update of Helsinki Declaration [25].

Patients who entered the study were over 18 years old, did not undergo scaling and root planing during the past 6 months and had CAL of ≥ 5 mm and PPD ≥ 6 mm with bleeding on probing around ≥ 2 non‐adjacent teeth [26].

Exclusion criteria: systemic diseases (e.g., uncontrolled diabetes mellitus, rheumatoid arthritis, osteoporosis), medications known to alter the immune response (e.g., chronic nonsteroidal anti-inflammatory drugs, glucocorticoids, bisphosphonates, calcitonin, methotrexate and antibiotics) affecting periodontal inflammation and bone turnover, paraphysiological conditions (e.g., pregnancy and breast feeding), and surgical periodontal therapy within the last year.

### Patient selection

The randomization was conducted using computer-generated sequencing. The sequence was placed in a sealed opaque envelope including the patient number and the treatment code. Patients were randomly assigned groups immediately after scaling and root planing. The practitioner (S.Y.), the examiner (H.A.) and the patient were blinded to group assignment. Informed consent was obtained from all subjects. According to amounts of α = 0.05, X_2_ = 9.3, X_1_ = 7.3 and β = 0.2, sample size set at n = 15 (standard deviation was considered 1.6 and 2.1 respectively).

### Clinical interventions

At the screening visit, the plaque index (PI) [27], gingivitis index (GI) [28], bleeding index (BI) [29], probing depth (PD), and clinical attachment level (CAL) were measured by an examiner (H.A) other than the practitioner who was blinded to the treatment assignment. All clinical parameters were measured on experimental teeth. The examiner was calibrated by “examiner alignment and assessment” protocol before measurements [30]. Intra-examiner reproducibility was assessed at the start of the study. All clinical measurements were performed twice for 5 patients (10 sites) with 24 h interval. The intra-examiner agreement was > 0.85. Indices were measured using a calibrated and standardized UNC-15 periodontal probe.

One day after the screening visit, oral hygiene instruction based on “targeted hygiene” was provided. Then, patients underwent full mouth supra- and subgingival scaling and root planning (SRP). The medications were applied immediately following the completion of the initial SRP, then repeated every other day for a total of 5 sessions. EPO is available as an ampule containing a solution of 4000 units [22]. One cc of EPO solution was mixed with 1 cc of carboxymethylcellulose gel to form an EPO gel at a concentration of 2000 units per cc. The resulting mixture was entirely (whole of 2 cc) applied in the test pockets (PPD ≥ 5 mm) using a 28-gauge needle. To ensure that EPO reached the desired depth, the tip of the canula was derived to the depth of the pocket. On the control sites, a placebo mixture of distilled water and carboxymethylcellulose gel, with the same volume of the test gel, was applied.

Patients were asked not to use any chemotherapeutic mouthwashes or other antibacterial or anti-inflammatory products. Patients were evaluated every two weeks for prophylaxis and oral hygiene reinforcement up to 3 months to prevent supra and sub- gingival plaque formation, and to keep the PI to 40% (based on the Sillness and Loe plaque index). Clinical indices were evaluated and measured by the same examiner at this time. In order to evaluate the need for future periodontal surgery, number of residual pockets with PPD ≥ 5 mm with bleeding upon probing was recorded.

### Statistical analysis

This study reports on the (3 month) change in clinical parameters between SRP + EPO and SRP only groups. We considered the BI and GI as primary outcomes and CAL, PD and PI as secondary outcomes. To determine the difference between the data before and after the treatment, as well as the differences in test and control groups data, the Wilcoxon Rank-Sum Test was used and the *P*-value below 0.05 was considered significant. The data were analyzed using SPSS software (Version 22.0, Chicago, IL, USA).

## Results

**Fig. 1:**
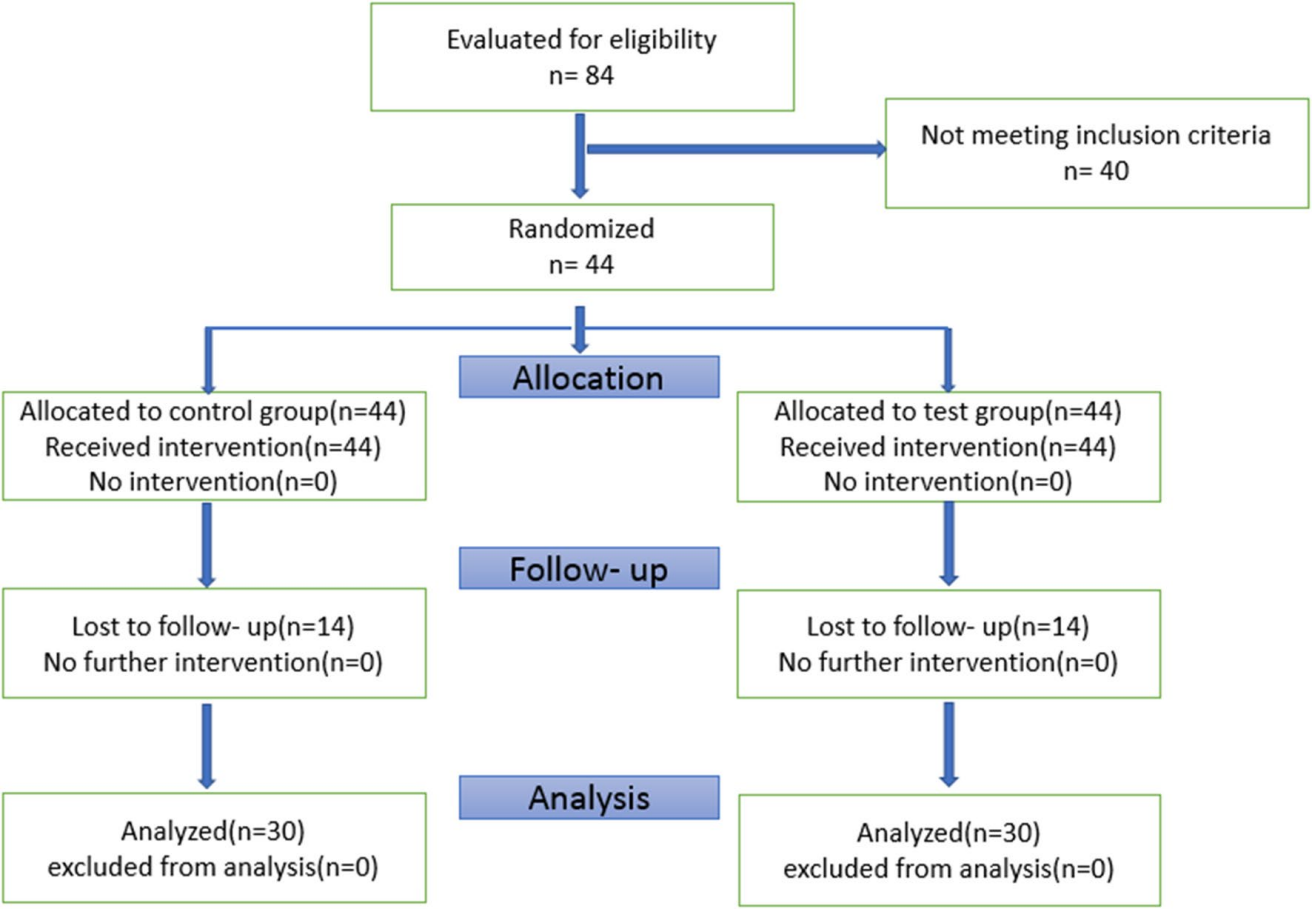
Consort flow chart

In this study, a total of 84 patients were examined, of which 44 were eligible to enter the study. Fourteen patients refused to continue their collaboration for various reasons, and finally, the study was conducted on 30 patients, 15 patients in each group (16 females and 14 males; age 32–68) (Table 1). In general, 60 teeth (30 in the experimental and 30 in the control groups) were included. None of the patients reported an allergic reaction to the drug or any other side effects. Consort flowchart is shown in Fig. 1.

**Table 1:** Baseline demographic data

Parameter	Test group (n = 15)	Control group (n = 15)
Age	32–68	32–68
Male/female	6/9	8/7
Teeth	30	30

At baseline, there were no significant difference in the scores of all of the clinical parameters between test and control groups (*P* > 0.05) (Table 2).

**Table 2:** Clinical parameters at baseline and 3 months post treatment

	Baseline		Post-treatment		*P*-value (Between- group comparison)
	Test	Control	Test	Control	
Plaque index (PI)	2.53 ± 0.89^αμ^	2.71 ± 0.46^αμ^	1.1 ± 0.79^α^	1.1 ± 0.68^α^	0.705
Gingival Index (GI)	2.6 ± 0.72^μ^	2.75 ± 0.75^μ^	0.98 ± 0.58^β^	1.33 ± 0.07^β^	0.002
Bleeding Index (BI)	2.71 ± 0.65^μ^	2.53 ± 0.89^μ^	0.84 ± 0.48^β^	1.3 ± 0.79^β^	0.000
Probing depth (mm)	3.72 ± 0.72^μ^	3.94 ± 0.78^μ^	1.95 ± 0.76^β^	2.55 ± 0.86^β^	0.004
CAL (mm)	5.1 ± 4.1^μ^	5.67 ± 4.32^μ^	3.40 ± 2.71^β^	4.33 ± 3.19^β^	0.002

^μ^No statistically significant difference between groups in baseline measurements (*p* > 0.05)

^α^PI was not significantly different between groups at any time points

^β^GI, BI, PD and CAL were significantly different between groups after 3 months

After 3 months, the GI scores decreased to 0.98 ± 0.58 and 1.33 ± 0.07 in test and control groups respectively. When compared between groups, the difference between two groups was significant (*P*-value = 0.002). At 3 months recall visit, BI scores decreased significantly in both groups (0.84 $\pm$0.48 in test and 1.3 $\pm$0.89 in control group). Comparison between groups revealed a significant difference between groups (*P*-value = 0.000). PI decreased in both groups without significant difference between groups (*P*-value = 0.705) (Table2).

In the post-treatment examinations, the PD values showed significant decrease within both study groups. When the comparison carried out between groups, there was a significantly greater decrease in PD in the test group (*P*-value = 0.004). There was a significant improvement in CAL values after treatment in both groups, this improvement was statistically greater in the EPO treated group (*P*-value = 0.002) (Table2).

**Fig. 2:**
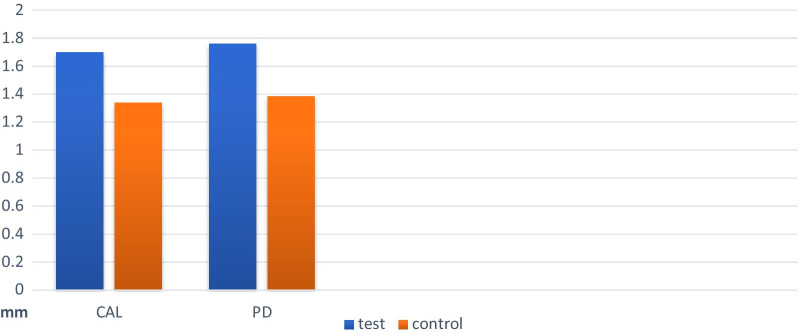
The amount of changes in periodontal parameters (CAL, PD) in each group during 3 months

**Fig. 3:**
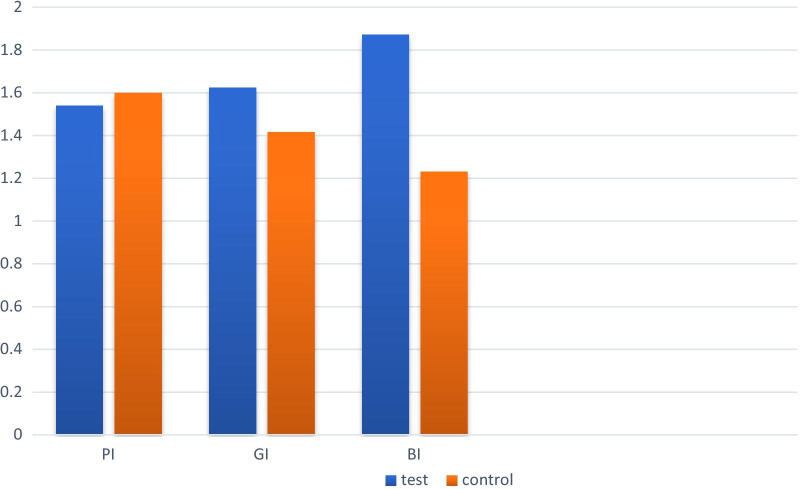
The amount of changes in clinical periodontal parameters (PI, BI, GI) in each group during 3 months follow up

Comparison of periodontal parameters at baseline and 3 months post treatment in test and control groups are presented in Figs. 2 and 3.

There was no significant difference in need for periodontal surgery. In all, 48% of EPO treated and 52% of control sites required surgical interventions (remaining pockets > 5 mm with BOP).

## Discussion

This study was aimed to investigate the efficacy of a topical erythropoietin for treatment of Stage III grade B/C periodontitis. After 3 months, the BI and GI scores (which reflecting the degree of gingival inflammation) decreased significantly in both groups. There was a statistically significant greater reduction in EPO treated group in comparison to patient received SRP alone.

Periodontal treatment primarily is intended to reduce the bacterial bioburden, by the mechanical removal of plaque and calculus (either by a nonsurgical, or surgical approach). Research into the pathogenesis of periodontal disease, particularly the role of the host response, has introduced opportunities for adjunctive pharmacotherapies in the management of periodontitis [2]. A variety of topical and systemically administered medications have been evaluated for their ability to modulate destructive components of the host response (e.g., Host Modulatory Therapies, HMTs) [31–33]. Although there is no study on the effectiveness of EPO on periodontal treatment, the positive effects of EPO on epithelial regeneration and healing process and its anti- inflammatory characteristic have been demonstrated [11, 18, 34]. Erythropoietin also exerts both cytoprotective and proangiogenic effects [12, 35, 36]. The osteogenic and angiogenic potential of erythropoietin are of particular interest for orthopedics [37]. Some studies showed that EPO improved bone healing and could serve as a therapeutic means enhancing bone regeneration [38, 39]. So, it was assumed that subgingival administration of EPO could be beneficial for its anti-inflammatory and regenerative properties in non- surgical periodontal therapies.

In 2004, Galeano and colleagues examined the effect of EPO on the extent of wound healing in diabetic rats on days 3, 6 and 12 after injury. They found that EPO had a significant effect on the level of angiogenesis, parallel with increased secretion of vascular endothelial growth factor (VEGF). VEGF is effective in both increasing the proliferation as well as in reducing the apoptosis of endothelial cells [40].

In a study by Fayaz-zadeh and colleagues in 2012, the effect of EPO and fibroblast growth factor (FGF2) on preventing the development of necrosis in skin flaps was investigated. Based on this study, both EPO treated and FGF2-treated groups had significantly less necrosis than control group, although this decrease was higher in FGF2 group [41]. They surveyed the magnitude of angiogenesis in all three groups (normal saline, EPO group, FGF-2 group) using a 400-magnification microscope and found no significant difference in the number of visible vessels in the three groups. This may means that less necrosis in the experimental groups is due to the improved microcirculation and increased number of small capillaries that are not visible with their assessment tool. Fayaz-zadeh and colleagues used EPO during different therapeutic periods. They observed that although high dose and frequent use of EPO did not make a significant difference in experimental group, the short-term (day 10) use with optimum dosage (100 U/kg/day) shows the best results [41].

In a systematic review, Hamed and colleagues studied the effect of EPO on wound healing of diabetic and non-diabetic animals and humans. The results showed that EPO was beneficial in the healing process of all these groups due to the effect of EPO on various stages of wound healing including the proliferative stage (the effect on fibroblastic proliferation and collagen synthesis) and the inflammatory stage (reduction of inflammatory cytokines and intrinsic immune cells). Bader and colleagues in 2011 studied the effect of EPO on the healing of foot skin lesions in humans. The result showed that EPO had a significant effect on the amount of epithelialization. In the EPO-treated group, epithelization was completed on the seventh post-treatment day while in the control group, the lesions were still red and exudating [19]. Our data showed that CAL and PD decreased in both groups after treatment, but the decrease in the experimental group was higher and there was a statistically significant difference between experimental and control group 3 months post treatment.

The cytoprotective effects of EPO (increased proliferation of fibroblasts and synthesis of collagen, increased number of macrophages, stimulated angiogenesis and reduced inflammatory response) and the expression of EPO receptors on the basal cells of the oral mucosa, could be assumed as the potential underlying mechanisms for greater reduction in BI and GI after non-surgical periodontal therapy [18].

To date, conventional treatments have succeeded in controlling periodontal inflammation but they had limited potentials toward regeneration of tissues destroyed during active periodontal disease [42].

Several studies have suggested that EPO can trigger bone formation from mesenchymal stem cells (mScs) [43, 44]. Kim et al., reported that EPO can regulate differentiation of both osteoblasts and osteoclasts through mechanistic target of rapamycin kinase (mtor) signaling. In this process, EPO improved bone formation of mScs [44]. In the study by Hamed, investigators reported that EPO promotes healing and stimulate the synthesis of collagen [45]. Accordingly, the increased clinical attachment gain could be attributed to the effects of EPO on bone formation and epithelial attachment.

Reduced gingival inflammation secondary to mechanical control of microbial biofilm and irritants promotes establishment of long junctional epithelium and consequently, a new periodontal attachment [46]. In addition, decreased penetration of periodontal probe may be a result of reduced tissue inflammation [47]. In a 12 weeks experimental study by Omlor etal, authors concluded that systemic or locally administration of EPO, as an additional treatment option, was beneficial to increase bone healing in rabbit long- bone defects. In addition administration of EPO significantly increased blood vessel formation after either local of systemic single-dose EPO treatment [48]. Therefore, the higher reduction in PD may be a result of reduced inflammatory response, gingival recession and bone regeneration in EPO treated sites.

Several studies showed the beneficial effect of administration of sub anti- microbial dose Doxycycline (SDD) [49, 50] and host modulatory drugs [51] on periodontal parameters improvement which could reduce the need for surgical interventions. In our study, the difference in the percentage of patients requiring surgery (PD > 5 mm) between the experimental and the control group was not statistically significant at the end of the study, which implicates further studies designed to investigate different doses of EPO within shorter intervals on larger amount of patients [41].

Locally delivered, controlled-release antimicrobials have been designed to maintain high and clinically relevant concentrations of drug in the GCF for extended periods [10]. Administration of controlled-release EPO may have more desirable effects. These systems can maximize the clinical benefit for patients. In addition, with controlled-release systems, the medication is applied in fewer visits with longer intervals. So, the main limitation of this study is that EPO was not formulated in a controlled-release gel. In order to improve the drug effectiveness, the EPO gel was applied 5 times every other day for 10 days.

Topical erythropoietin may increase the plasma erythropoietin concentration that can cause hematological changes and result in systemic side effects [48]. However, in our study blood samples were not collected to determine the plasma erythropoietin levels and blood cell counts before and after the topical administration of the drug. So, we are unable to conclude that this treatment modality is systemically safe.

## Conclusion

Considering the limitations of this study, the results showed that topical administration of EPO has no beneficial effect on the reduction of microbial plaque. However, when used as an adjunct to non-surgical periodontal therapy, it provides a promising result in treatment of patients with moderate to severe chronic periodontitis. Since in this study only three-month follow ups were conducted, we suggest further studies with longer follow up periods. Moreover, we suggest further studies on larger number of subjects and with various doses of EPO to determine the optimal dose of the drug.

## Supplementary Information

**Additional file 1**. Ethics-informed consent.

## Data Availability

Data and materials are available with corresponding author.

## Abbreviations

SRP: Scaling and root planning
EPO: Erythropoietin
PI: Plaque index
GI: Gingival index
CAL: Clinical attachment level
PD: Probing depth
BI: Bleeding index
HMTs: Host modulatory therapies
VEGF: Vascular endothelial growth factor
FGF2: Fibroblast growth factor
mScs: From mesenchymal stem cells
mtor: Mechanistic target of rapamycin kinase
SDD: Sub anti- microbial dose doxycycline
