# Supplementary material for: The effects of topical erythropoietin on non-surgical treatment of periodontitis: a preliminary study
Source: BMC Oral Health. 2021 May 6;21:240. doi: 10.1186/s12903-021-01607-y (PMC8101234; doi:10.1186/s12903-021-01607-y)
Supplement: Supplementary file 1 — Additional file 1. Ethics-informed consent. [file 12903_2021_1607_MOESM1_ESM.doc]

**Informed Consent form for patients who inviting to participate in research titled: ”** **The Effects of Topical Erythropoietin on Non- surgical Treatment of Periodontitis: A Preliminary Study”**

**at Tehran University of medical sciences, Faculty of Dentistry**

**[Name of Principal Investigator: Hoori Aslroosta]**

**[Name of Organization: Tehran University of medical sciences]**

**This Informed Consent Form has two parts:**

- **Information Sheet (to share information about the research with you)**
- **Certificate of Consent (for signatures if you agree to take part)**

**You will be given a copy of the full Informed Consent Form**

**PART I: Information Sheet**

**Introduction**

I am Dr. Hoori Aslroosta, working for the Tehran University of medical sciences(TUMS) Research Institute. We are doing research on periodontitis disease, which is very common in this country. I am going to give you information and invite you to be part of this research. You do not have to decide today whether or not you will participate in the research. Before you decide, you can talk to anyone you feel comfortable with about the research.

*There may be some words that you do not understand. Please ask me to stop as we go through the information and I will take time to explain. If you have questions later, you can ask them of me, the study doctor or the staff*

**Purpose of the research**

This research will involve a scaling and root planning procedure followed by an injection for a total of 5 sessions in the periodontal pockets as well as follow-up visits

**Participant selection**

We are inviting all adults with stage III periodontitis who TUMS to participate in the research.

**Voluntary Participation**

Your participation in this research is entirely voluntary. It is your choice whether to participate or not. Whether you choose to participate or not, all the services you receive at TUMS will continue and nothing will change. If you choose not to participate in this research project, you will offered the treatment that is routinely offered in TUMS for disease.

- *If you decide not to take part in this research study, do you know what your options are? Do you know that you do not have to take part in this research study, if you do not wish to? Do you have any questions?*

**Description of the Process**

During the research you make 12 visits.

- In the first visit, some indexes measured by an examiner and scaling- root planning will be done.
- At the next visit, we start drug injection for a total of 5 sessions.
- You will be evaluated every two weeks for prophylaxis and oral hygiene reinforcement up to 3 months.

**Sharing the Results**

The knowledge that we get from doing this research will be shared with you through community meetings before it is made widely available to the public. Confidential information will not be shared. There will be small meetings in the community and these will be announced. After these meetings, we will publish the results in order that other interested people may learn from our research.

**Right to Refuse or Withdraw**

You do not have to take part in this research if you do not wish to do so. You may also stop participating in the research at any time you choose. It is your choice and all of your rights will still be respected.

**Alternatives to Participating**

If you do not wish to take part in the research, you will be provided with the established standard treatment available at TUMS.

**Who to Contact**

If you have any questions you may ask them now or later, even after the study has started. If you wish to ask questions later, you may contact any of the following: [Hoori Aslroosta. 02188015950]

**This proposal has been reviewed and approved by TUMS, which is a committee whose task it is to make sure that research participants are protected from harm.**

**PART II: Certificate of Consent**

**I have read the foregoing information, or it has been read to me. I have had the opportunity to ask questions about it and any questions that I have asked have been answered to my satisfaction. I consent voluntarily to participate as a participant in this research.**

**Print Name of Participant__________________**

**Signature of Participant ___________________**

**Date ___________________________**

**Day/month/year**

**If illiterate**

A literate witness must sign (if possible, this person should be selected by the participant and should have no connection to the research team). Participants who are illiterate should include their thumb-print as well.

**I have witnessed the accurate reading of the consent form to the potential participant, and the individual has had the opportunity to ask questions. I confirm that the individual has given consent freely.**

**Print name of witness_____________________ AND Thumb print of participant**

**Signature of witness ______________________**

**Date ________________________**

**Day/month/year**

**Statement by the researcher/person taking consent**

**I confirm that the participant was given an opportunity to ask questions about the study, and all the questions asked by the participant have been answered correctly and to the best of my ability. I confirm that the individual has not been coerced into giving consent, and the consent has been given freely and voluntarily.**

**A copy of this ICF has been provided to the participant.**

**Print Name of Researcher****/person taking the consent________________________**

**Signature of Researcher /person taking the consent__________________________**

**Date ___________________________ Day/month/year**
